# Electroacupuncture reduces oxidative stress response and improves secondary injury of intracerebral hemorrhage in rats by activating the peroxisome proliferator-activated receptor-γ/nuclear factor erythroid2-related factor 2/γ-glutamylcysteine synthetase pathway

**DOI:** 10.1097/WNR.0000000000002026

**Published:** 2024-04-08

**Authors:** Weigang Luo, Wei Bu, Hequn Chen, Wanhu Liu, Xudong Lu, Guisong Zhang, Cuicui Liu, Xiaohui Li, Huiling Ren

**Affiliations:** a Department of Neurology; b Department of Neurosurgery, The Third Hospital of Hebei Medical University; c Basic Medical College, Hebei Medical University, Shijiazhuang, China

**Keywords:** γ-GCS, electroacupuncture, intracerebral hemorrhage, microglia, Nrf2, PPARγ

## Abstract

Intracerebral hemorrhage (ICH) is a severe stroke subtype. Secondary injury is a key factor leading to neurological deficits after ICH. Electroacupuncture (EA) can improve the neurological function after ICH, however, its internal mechanism is still unclear. The aim of this study is to investigate whether EA could ameliorate secondary injury after ICH through antioxidative stress and its potential regulatory mechanism. A rat model of ICH was established by injecting autologous blood into striatum. After the intervention of EA and EA combined with peroxisome proliferator-activated receptor-γ (PPARγ) blocker, Zea-longa scores, modified neurological severity scores and open field tests were used to evaluate the neurological function of the rats. Flow cytometry detected tissue reactive oxygen species (ROS) levels. Tissue tumor necrosis factor-α (TNF-α) levels were analyzed by enzyme-linked immunosorbent assays. The protein expressions of PPAR γ, nuclear factor erythroid2-related factor 2 (Nrf2) and γ-glutamylcysteine synthetase (γ-GCS) were detected by Western blot. Immunohistochemistry was used to observe the activation of microglia. The demyelination degree of axon myelin was observed by transmission electron microscope. Compared with the model group, EA intervention improved neurological function, decreased ROS and TNF-α levels, increased the protein expression of PPARγ, Nrf2 and γ-GCS, and reduced the activation of microglia, it also alleviated axonal myelin sheath damage. In addition, the neuroprotective effect of EA was partially attenuated by PPARγ blocker. EA ameliorated the neurological function of secondary injury after ICH in rats, possibly by activating the PPARγ/Nrf2/γ-GCS signaling pathway, reducing microglia activation, and inhibiting oxidative stress, thus alleviating the extent of axonal demyelination plays a role.

## Introduction

Intracerebral hemorrhage (ICH) is the most fatal form of acute stroke, with 17.9 million patients worldwide, accounting for 10–15% of all stroke patients [1]. Despite treatments such as surgical hematoma evacuation and blood pressure management, mortality from ICH remains as high as 68% [1]. ICH-induced injuries are classified into primary injuries and secondary injuries. The primary injury was related to the mass effect caused by initial hematoma, hematoma expansion and hydrocephalus. Secondary brain injury is caused by microglia activation, which drives inflammatory, oxidative and cytotoxic reactions that further lead to cell death and dysfunction [2]. After ICH, reactive oxygen species (ROS) act as mediators of demyelination and blood-brain barrier disruption, causing mitochondrial dysfunction, and may be involved in the development of early and late brain edema. A recent multicenter clinical trial confirmed that early hematoma removal after ICH does not effectively improve patient prognosis [3]. In addition to limited progress in the management of ICH patients, effective treatment options are yet to be developed for the secondary brain injury of ICH.

Several studies have demonstrated that peroxisome proliferator-activated receptor-γ (PPARγ) can inhibit oxidative stress [4,5]. PPARγ can regulate the activation and differentiation of immune cells, thereby regulating the secretion of inflammatory factors and reducing the production of ROS [6]. PPARγ can also regulate the nuclear factor erythroid2-related factor 2 (Nrf2)/antioxidant responsive element pathway, regulate downstream gene expression, and inhibit oxidative stress [6]. γ-glutamylcysteine synthetase (γ-GCS), as a downstream product of Nrf2, is the rate-limiting enzyme of glutathione (GSH) biosynthesis and an important part of antioxidant activity [7].

More and more basic experiments have demonstrated that electroacupuncture (EA) can improve the neurological deficits and brain damage of ICH, but the underlying mechanisms remain unclear and deserve further investigation [8,9]. In this study, we investigated whether EA could attenuate the secondary brain injury induced by oxidative stress after ICH. To further explore the potential mechanism of neuroprotective effect of EA after ICH and provide a new target for the treatment of ICH.

## Materials and methods

### Animals

All animal experiments were approved by the Experimental Animal Ethics Committee of the Third Hospital of Hebei Medical University (Approval No.2022-020-1). Male Sprague–Dawley rats (250–280 g) were provided by Vital River Laboratory Animal Technology Co., Ltd., Beijing, China. Food and water were provided ad libitum. Rats were housed under a 12-h light/dark cycle with room temperature, with the lights on and off at 7:00 and 19:00, respectively. The temperature was 25 ± 2 °C and the humidity was 50 ± 5%.

### Intracerebral hemorrhage model

A rat ICH model was constructed using intracerebral injection of autologous blood as described in a previous study [10]. Rats were anesthetized with intraperitoneal injection of sodium pentobarbital (45 mg/kg) and placed in a stereotaxic frame. After routine disinfection and incision, a dental drill is used to make a hole in the skull. Autologous blood (100 μl) was injected with a microsyringe into the striatum 3.3 mm posterior to bregma, 3.1 mm lateral to the right side of the sagittal suture and 8 mm downward from the vertical skull surface. Then, the skull hole was sealed with bone wax, disinfected and sutured and the rat was removed from the stereotaxic frame. Rats in the sham group were injected with the same amount of normal saline, and other operations were the same as those in the ICH group.

### Experimental design

The 40 rats were randomly divided into a sham group (*n* = 10) and model reserve group (*n* = 30). Among the 30 rats, 27 rats were successfully modeled, and were further randomly divided into three groups: model group, EA group, and EA+blocker group. The EA group started EA intervention on the second day after ICH modeling. The Quchi acupoint is selected in the depression in front of the outer side of the elbow joint at the proximal end of the radius, and it is punctured 4 mm in a straight line; The Zusanli acupoint is selected at the place about 5 mm below the head of the fibula at the outer lower part of the knee joint of the hind limb, and the point is pierced 7 mm. Rats were restrained in the supine position, routinely disinfected, acupunctured with sterile acupuncture needles, and twisted 90–180°; an SDZ-V EA therapy instrument was used for intervention. EA intervention parameters: density wave, 100 Hz, current intensity 1–3 mA, stimulation intensity is suitable for slight shaking of rat limbs, 30 min each time, once a day, for seven consecutive days. The EA+blocker group first received an intraperitoneal injection of T0070907 (2 mg/kg/day), and one hour after the injection, anesthesia and restraint were performed by intraperitoneal injection of sodium pentobarbital (45 mg/kg). The same EA intervention as the EA group was performed, with intraperitoneal injection and EA once a day for seven consecutive days. The sham group and the model group were given the same anesthesia restraint. The experimental procedures are listed in Table 1.

**Table 1:** Experimental operation process

Period	The first day	The second day to the eighth day	The ninth day
Sham	Saline injection into striatum	Anesthesia and restraint	Neurologic score, open field test, and collection of brain tissue
Model	Autologous blood injection into striatum	Anesthesia and restraint	Neurologic score, open field test, and collection of brain tissue
EA	Autologous blood injection into striatum	EA intervention for 30 min after anesthesia and restraint	Neurologic score, open field test, and collection of brain tissue
EA+blocker	Autologous blood injection into striatum	One hour after the injection of blocker, the rats were anesthetized and restrained and electroacupuncture intervention was performed for 30 min	Neurologic score, open field test, and collection of brain tissue

### Zea-longa scores

Neurological deficits in rats after ICH were assessed according to the Zea-longa score [11]. (1) no neurological injury symptoms; (2) contralateral forepaws of the rats cannot stretch freely (mild neurological deficits); (3) show contralateral rotation during walking (moderate neurological deficit); (4) show contralateral falling during walking (severe neurological deficits); and (5) fail to walk spontaneously and show loss of consciousness. Rats were scored for neurological function after they woke up, and a score of 1–3 indicated that the model was successfully included in the follow-up study. Scores were repeated 7 days after ICH.

### Modified neurological severity scores

Neurological deficits were evaluated using the modified neurological severity scores (mNSS), consisting of tests on sensation, movement, balance and reflexes. Detailed operations of mNSS include: raising rat by the tail, placing rat on the floor, sensory tests, beam balance tests and reflexes absent and abnormal movements. Its score ranged from 0 to 18:0, no deficit; 1–6, mild deficit; 7–12, moderate deficit; and 13–18, severe deficit. Higher scores indicate worse neurological function [12]. A researcher who was unaware of the experimental grouping performed mNSS at 1 and 7 days after ICH.

### Open field test

Open field test (OFT) was used to assess spontaneous activity and exploratory behavior in rats. Record the rat’s movement distance, movement speed, and resting time within 15 min, and record the video synchronously. The experimental chamber (black, square, length × width × height = 100 cm × 100 cm × 40 cm) was divided into peripheral area and central area. The rat open field experimental box was evenly divided into 16 square areas, the four squares in the middle were the central area, and the surrounding 12 squares were the peripheral areas. Camera system and animal behavior video analysis software VisuTrack (Shanghai Xinruan Information Technology Co., Ltd., Shanghai, China) were used to record and analyze the pontaneous activities of rats.

### Reactive oxygen species detection

After deep anesthesia by sodium pentobarbital (45 mg/kg i.p.), the cortex tissue around right ICH was extracted. The flow cytometry was used to determine the levels of intracellular ROS using Reactive Oxygen Species Assay Kit (S0033S, Beyotime Biotechnology, Shanghai, China) following the manufacturer’s instructions. The collected cells were suspended in 2',7'-dichlorodihydrofluorescein diacetate at a concentration of 10 µM/l, and incubated in a cell culture incubator at 37 °C for 20 min. Cells were washed three times with serum-free medium and analyzed by flow cytometry (Beckman, FC500).

### Enzyme-linked immunosorbent assays

After deep anesthesia by sodium pentobarbital (45 mg/kg i.p.), the cortex tissue around right ICH was extracted. Homogenates of cortical tissue around the injury site were taken, centrifuged (12 000 × g for 20 min at room temperature), and the supernatant obtained. Expression of tumor necrosis factor-α (TNF-α) was measured using the kits (SEA133Ra, Cloud-Clone, Wuhan, China).

### Western blotting

After deep anesthesia by sodium pentobarbital (45 mg/kg i.p.), the cortex tissue around right ICH was extracted. Homogenize the tissue samples in radio immunoprecipitation assay buffer containing a protease inhibitor cocktail. Protein concentration was measured with a BCA Protein Assay Kit (No.23228, Thermo, Waltham, Massachusetts, USA). Proteins were separated by 10% SDS-Page electrophoresis and transferred to polyvinylidene fluoride (PVDF) membranes, then PVDF membranes were blocked with 5% skimmed milk for 1 h, primary antibodies were added and incubated overnight in a 4 °C refrigerator. The primary antibodies used were as follows: anti-PPARγ (1:1000), anti-Nrf2 (1:4000), anti-γ-GCS(1:3000) and anti-β-Actin(1:1000). Membranes were washed and incubated with goat anti-rabbit antibody (1:3000) for 2 h at 37 °C. The proteins were visualized by chemiluminescence reagents (ECL, Amersham). Grayscale analysis of protein bands was performed using ImageJ, and β-Actin was used as a quantitative control.

### Immunohistochemical staining

After the rats were anesthetized by sodium pentobarbital (45 mg/kg i.p.), the heart was perfused with 4% paraformaldehyde, and the brain tissue was extracted. Tissues were fixed with 4% paraformaldehyde, sectioned, deparaffinized with xylene, and hydrated with ethanol. The brain tissue sections were placed in anti-Iba1 primary antibody (1:200) overnight at 4 °C, and washed three times with PBS. Biotin-labeled goat anti-rabbit Immunoglobulin G((1:1000) secondary antibody was added to the sections and incubated at 37 °C for 30 min. Sections were then washed with PBS and incubated with avidin–peroxidase complex for 30 min before the immunocomplex was visualized using the chromogen 3,3-diaminobenzidine. After counterstaining with hematoxylin and dehydration, sections were cleared in xylene and covered with neutral balsam. Take pictures under a light microscope. The regions of interest were the motor cortex on the right side of the sections, and the start of the sections used for cell counting was 3.30 mm behind bregma, with a spacing of 4 μm. ImageJ software was used to detect the number of activated microglia and calculate the density of activated microglia across the image area, with analysis units of piece/mm^2^.

### Transmission electron microscopy

After the rats were anesthetized by sodium pentobarbital (45 mg/kg i.p.), the heart was perfused with glutaraldehyde fixative solution and the brain tissues were taken. The tissue of 1 mm×1 mm×3 mm taken from the cerebral cortex was fixed by immersing in 4% glutaraldehyde solution for more than 2–4 h. The tissues were then fixed with 1% osmium tetroxide for 2 h. Tissue samples were dehydrated with gradient acetone, then embedded, put into an oven for polymerization, stored at 37 °C for 24 h, and then placed at 60 °C for 48 h. Afterwards, the sample was cut into ultrathin slices with a thickness of about 50 nm with an ultramicrotome, and double-stained with uranyl acetate and lead citrate. Finally, the morphology and ultrastructure of the axons and myelin sheaths of neurons were observed under a transmission electron microscope (JEOL JEM-1230). There are three rats in each group, and each rat has three photos, for a total of 36 photos. The g-ratio is used to assess the extent of axonal demyelination and is calculated by dividing the axonal diameter by the diameter of the entire nerve fiber [13]. ImageJ software measures the maximum and minimum diameters, and then calculates the average of the two for analysis.

### Statistical analysis

All data are presented as mean ± SD and analyzed using SPSS 25.0 software package (Chicago, Illinois, USA). Multigroup comparisons were performed with one-way analysis of variance. Multiple post hoc comparisons were then performed using the least significant difference test. Statistical significance was set at *P* < 0.05.

## Results

### Electroacupuncture improved neurological function in rats after intracerebral hemorrhage

**Fig. 1:**
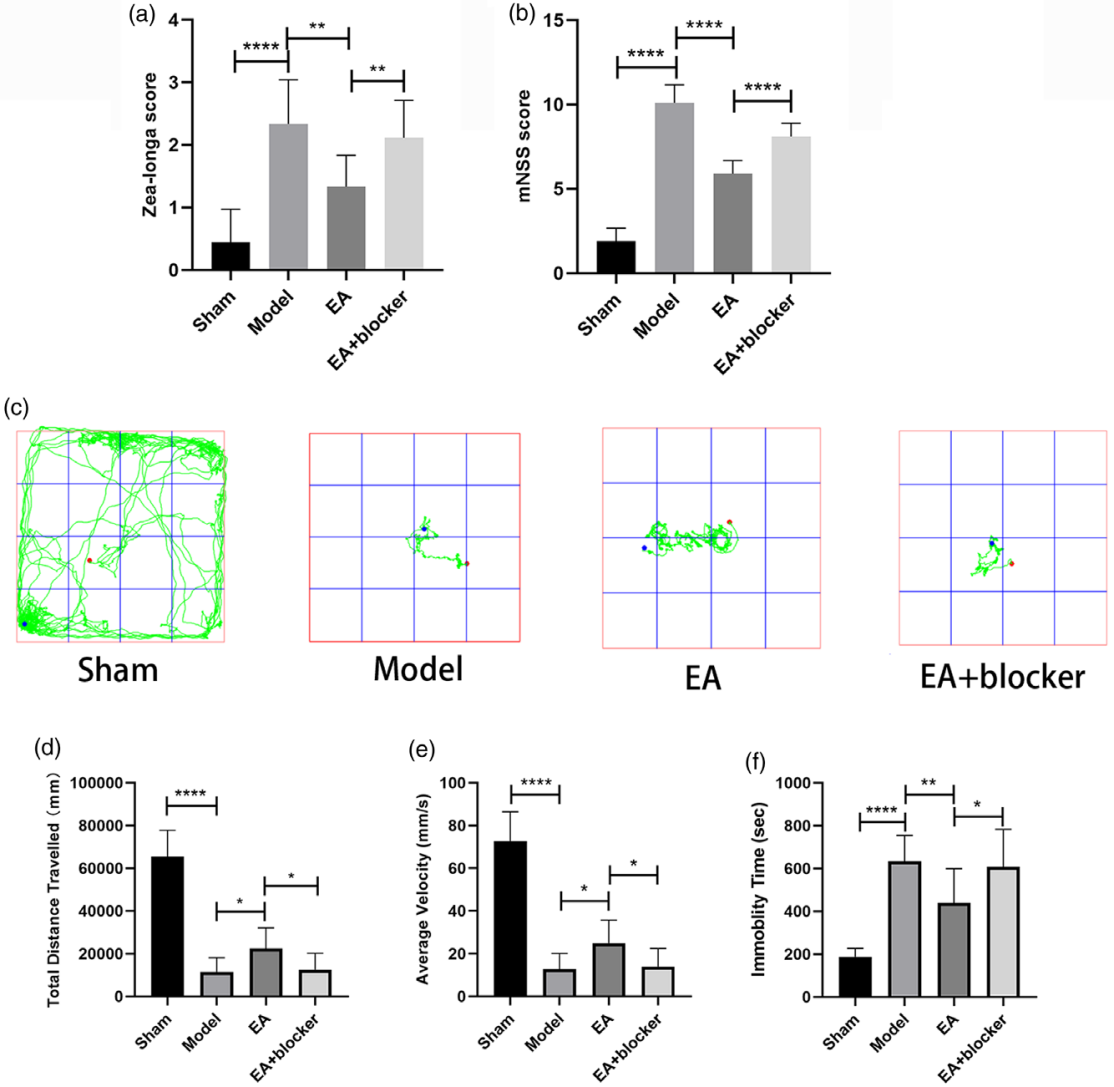
Electroacupuncture improved neurological function in rats after intracerebral hemorrhage. (a) Analysis of Zea-longa scores for each group. (b) Analysis of mNSS scores for each group. (c) Open field trajectory diagram of rats in each group. (d, e and f) Analysis of the total distance traveled, average velocity and immobility time of rats in each group in the open field test. The results are presented as the mean ± SD of nine independent experiments. *P < 0.05; **P < 0.01; ****P < 0.0001. mNSS, modified neurological severity scores.

The Zea-longa score [*F*(3,32) = 18.987, *P* < 0.001] and mHSS score [*F*(3,32) = 151.899, *P* < 0.001] of the model group were significantly higher than those of the sham group; Compared with the model group, the Zea-longa score and mHSS score of the EA group were significantly decreased; The Zea-longa score and mHSS score of the EA+blocker group were higher than those of the EA group (Fig. 1a, b). In the OFT, the total distance traveled [*F*(3,32) = 66.599, *P* < 0.001] and average velocity [*F*(3,32) = 66.908, *P* < 0.001] of the model group were significantly lower than those of the sham group, and the immobility time [*F*(3,32) = 20.987, *P* < 0.001] was significantly higher than that of the sham group; Compared with the model group, the total distance traveled and average velocity of the EA group increased, and the immobility time decreased; The total distance traveled and average velocity of the EA+blocker group were lower than those of the EA group, and the immobility time was higher than that of the EA group (Fig. 1c–f).

### Electroacupuncture inhibited oxidative stress after intracerebral hemorrhage in rats

**Fig. 2:**
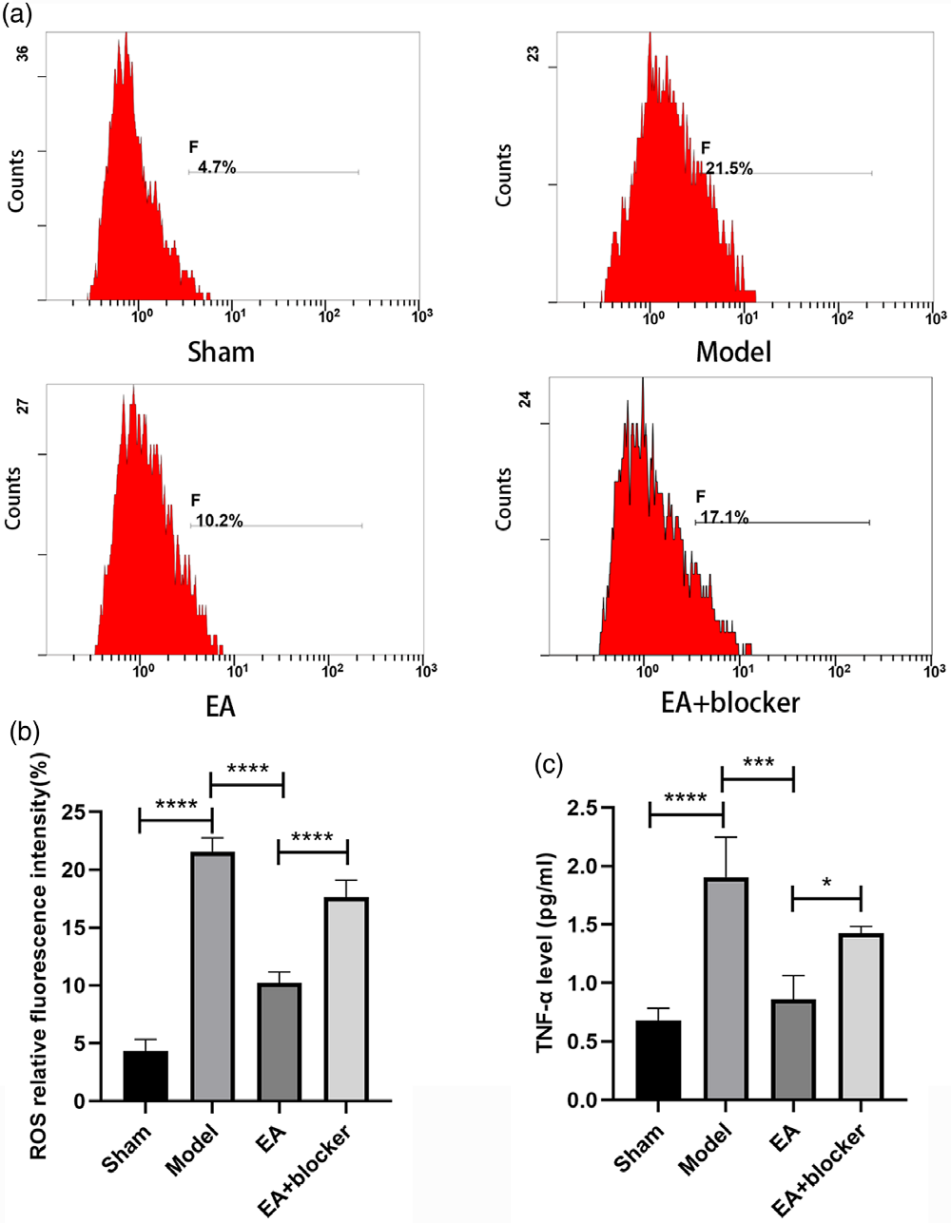
Electroacupuncture inhibited oxidative stress and inflammatory responses after cerebral hemorrhage in rats. (a) Flow cytometry of rat tissue cells in each group. (b) Analysis of relative fluorescence intensity of ROS in each group. (c) Analysis of TNF-α levels in each group. The results are presented as the mean ± SD of three independent experiments. *P < 0.05; ***P < 0.001; ****P < 0.0001. ROS, reactive oxygen species; TNF-α, tumor necrosis factor-α.

The relative fluorescence intensity of ROS [*F*(3,8) = 128.068, *P* < 0.001] in the model group was significantly higher than that in the sham group; compared with the model group, the relative fluorescence intensity of ROS in the EA group was significantly reduced; The relative fluorescence intensity of ROS in the EA+blocker group was higher than that in the EA group (Fig. 2a, b). The TNF-α level [*F*(3,8) = 21.536, *P* < 0.001] in the model group was significantly higher than that in the sham group; compared with the model group, the TNF-α level in the EA group was significantly reduced; the TNF-α level in the EA+blocker group was higher than that in the EA group (Fig. 2c).

### Electroacupuncture increased the expressions of peroxisome proliferator-activated receptor-γ, nuclear factor erythroid2-related factor 2 and γ-glutamylcysteine synthetase in the perihematoma tissue after intracerebral hemorrhage in rats

**Fig. 3:**
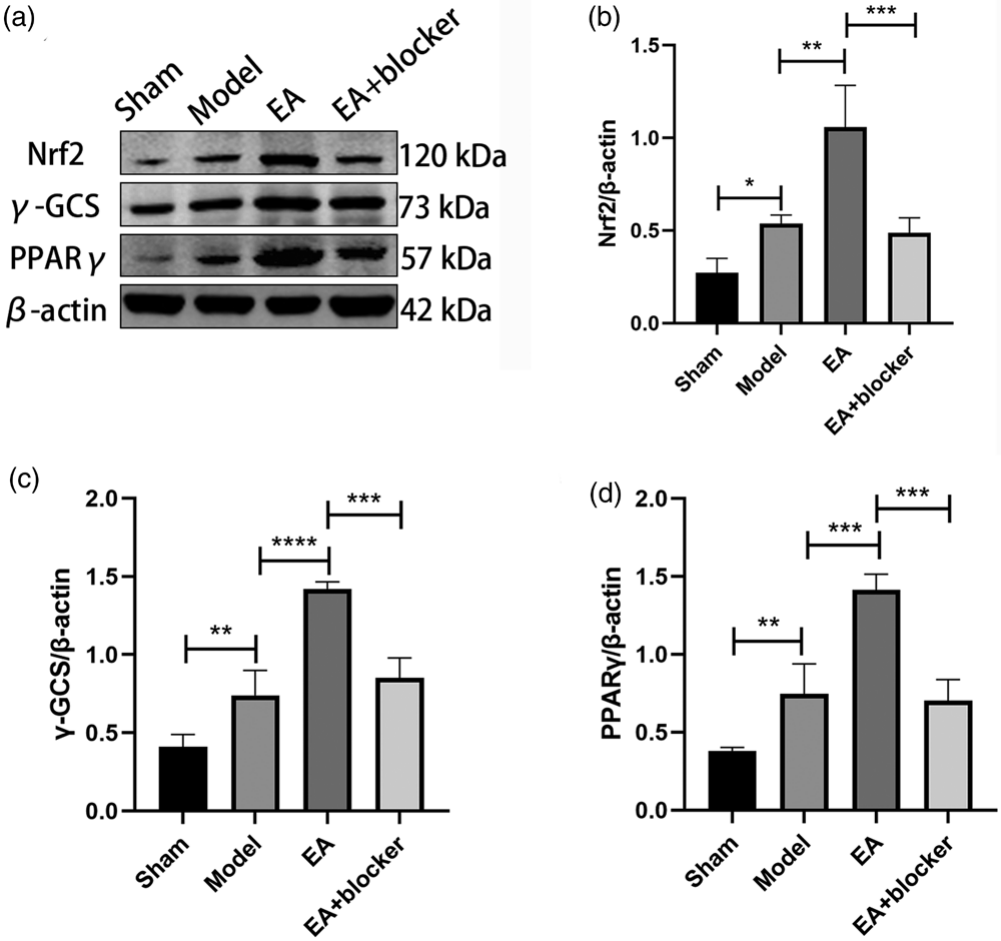
Electroacupuncture increased the expressions of PPARγ, Nrf2 and γ-GCS in the perihematoma tissue after ICH in rats. (a) Western blotting method was used to detect the expression of PPARγ, Nrf2 and γ-GCS in the tissues around the hematoma after ICH. (b) The relative expression of Nrf2 was quantified by normalizing it to β-actin. (c) The relative expression of γ-GCS was quantified by normalizing it to β-actin. (d)The relative expression of PPARγ was quantified by normalizing it to β-actin. The results are presented as the mean ± SD of three independent experiments. *P < 0.05; **P < 0.01; ***P < 0.001; ****P < 0.0001. γ-GCS, γ-glutamylcysteine synthetase; Nrf2, nuclear factor erythroid2-related factor 2; PPARγ, peroxisome proliferator-activated receptor-γ.

Compared with the sham group, the contents of PPARγ [*F*(3,8) = 41.711, *P* < 0.001], Nrf2 [*F*(3,8) = 20.360, *P* < 0.001] and γ-GCS [*F*(3,8) = 33.926, *P <* 0.001] in the model group were significantly increased. The contents of PPARγ, Nrf2 and γ-GCS in the EA group were higher than those in the model group. Compared with the EA group, the PPARγ, Nrf2 and γ-GCS contents in the EA+blocker group were decreased (Fig. 3a–d).

### Electroacupuncture inhibits microglial activation after intracerebral hemorrhage in rats

**Fig. 4:**
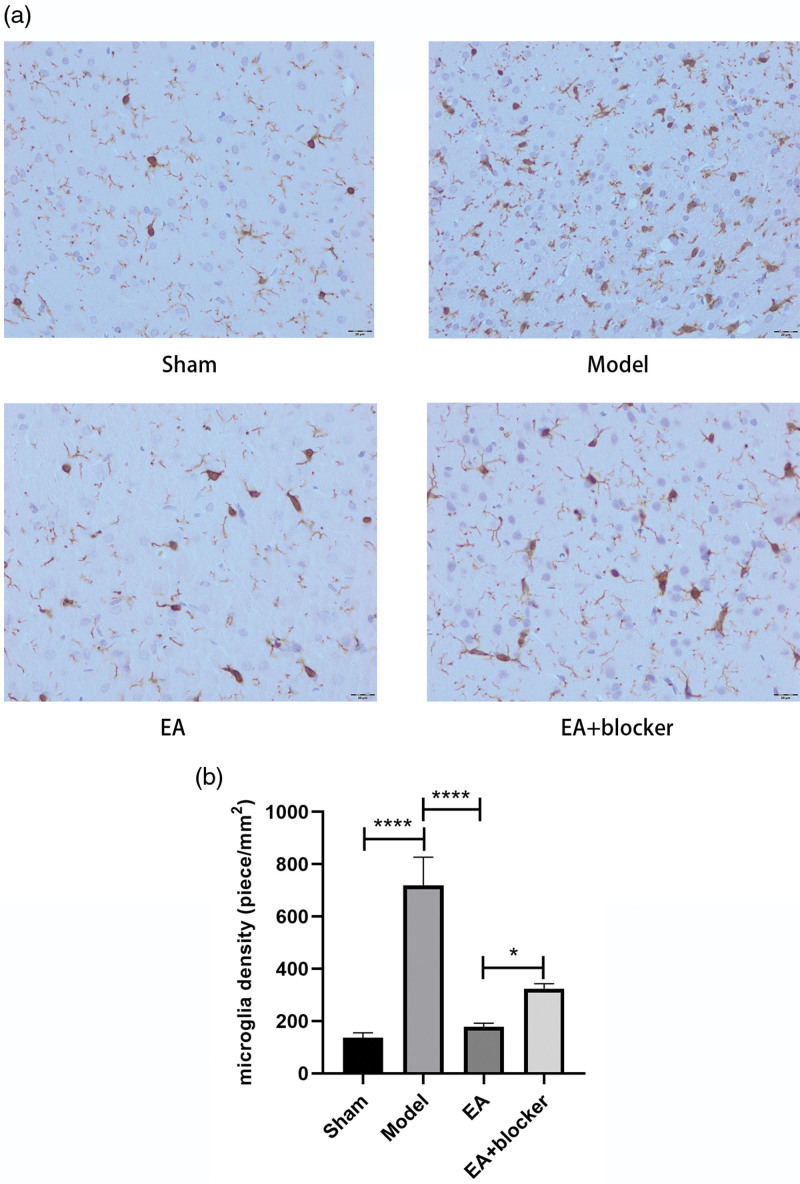
Electroacupuncture inhibited the activation of microglia in perihematoma tissue. (a) Immunohistochemistry of microglia. (b) Analysis of microglia density in each group. The results are presented as the mean ± SD of three independent experiments. *P < 0.05; ****P < 0.0001.

Compared with the sham group, the density of activated microglia [*F*(3,8) = 68.332, *P* < 0.001] in the perihematoma tissue of the model group was significantly higher. EA had significantly inhibited the activated microglia in the perihematoma tissue compared to the model group. The density of activated microglia in the EA+PPARγ blocker group was higher than that in the EA group (Fig. 4a, b).

### Electroacupuncture alleviated axonal demyelination injury after intracerebral hemorrhage in rats

**Fig. 5:**
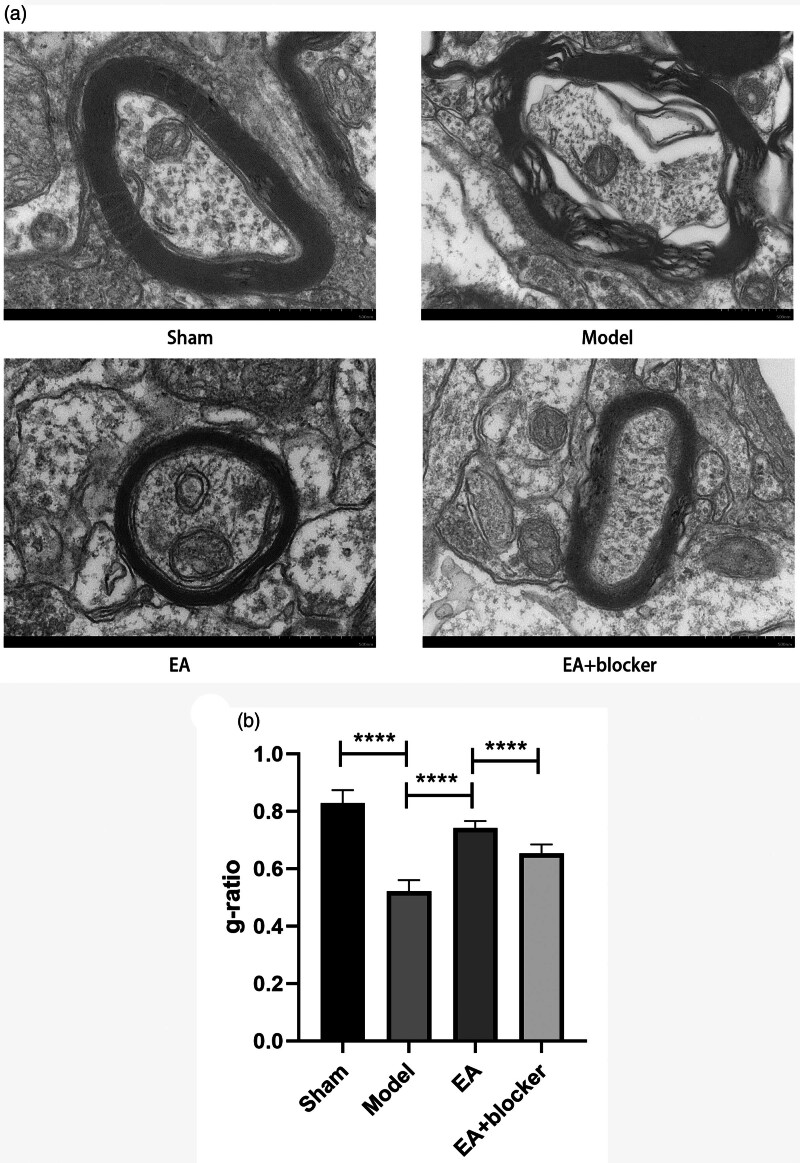
Electroacupuncture alleviated axonal demyelination injury after intracerebral hemorrhage in rats. (a) TEM of axonal myelin sheath in perihematoma tissue. (b) Analysis of g-ratio in each group. The results are presented as the mean ± SD of three independent experiments. ****P < 0.0001.

The axons of neurons in the sham group had uniform and complete circular plaques. Myelin tightly wraps the axons. The myelin on the axons of the model group was significantly damaged, the structure of the myelin sheath was uneven, and there were breaks. Compared with the model group, the injured degree of axon myelin sheath in the EA group was lighter, with less breakage and uniform structure. Compared with the EA group, the number of axonal myelin sheath injury sites in the EA+blocker group was more, and the degree of injury became more serious (Fig. 5a). Compared with the sham group, the g-ratio of the model group [*F*(3,26) = 98.413, *P* < 0.001] was significantly lower. Compared with the model group, the g-ratio in the EA group was significantly increased. The g-ratio of the EA+blocker group was lower than that of the EA group (Fig. 5a, b).

## Discussion

In clinical practice, acupuncture has been widely used in ICH patients and has been shown to significantly improve patients’ levels of consciousness, limb motor function, and cognitive deficits, among others [14–16]. With the common progress of acupuncture and evidence-based medicine, the clinical and basic researches on the treatment of ICH by EA continue to emerge, and the interpretation of its mechanism is also constantly improving. EA can exert neuroprotective effect on brain injury after ICH through many ways, such as anti-inflammation, antioxidation, antiapoptosis and increasing brain derived neurotrophic factor protein [17–19]. In this study, we found that EA could activate PPARγ/Nrf2/γ-GCS signaling pathway, inhibit microglia activation, reduce ROS production, and accelerate ROS clearance, thus alleviating oxidative stress response and the extent of myelin sheath damage, improve neurological function after ICH.

Oxidative stress is an imbalance between ROS production and the scavenging capacity of the antioxidant system. ROS accumulation after ICH plays an important role in secondary brain injury, resulting in lipid peroxidation, protein oxidation, inhibition of mitochondrial activity, and increased cell and lysosomal membrane permeability, eventually the cell swells/ruptures [20]. The important sources of ROS after ICH are mitochondrial dysfunction, erythrocyte degradation products, excitatory glutamate, activated microglia and infiltrated neutrophil [21]. These processes lead to mitochondrial Ca^2+^ overload after ICH, which reduces membrane potential and induces mitochondrial permeability transition pore opening. This destroys the mitochondrial respiratory chain and the oxidase nicotinamide adenine dinucleotide phosphate, resulting in the release of excess O_2_^−^ [22]. The results showed that ROS significantly increased and neurological function deteriorated after ICH, suggesting that oxidative stress is involved in secondary brain injury after ICH. After EA intervention, ROS was significantly decreased and neurological function was significantly improved, indicating that EA could play an antioxidative stress role and improve neurological function after ICH.

Microglia are central nervous system resident immune cells that monitor the central nervous system environment under physiological conditions and are key factors in the initiation of oxidative stress in ICH [23]. After ICH, various cytotoxic substances in intracranial hematomas activate microglia, and early microglia release large amounts of pro-inflammatory mediators [2]. TNF-α released by microglia, as the most important inflammatory factor, causes endothelial cell necrosis, thereby destroying the blood-brain barrier. Chemokines aggravate the infiltration and proliferation of neutrophils, causing apoptosis and necrosis of central neurons and glial cells, resulting in a large amount of ROS release, which is one of the main causes of secondary damage to brain tissue [24]. In stroke, over-activation of microglia leads to dysfunction of neuronal mitochondria, which in turn leads to autophagy, apoptosis or necrosis of neurons [25]. Therefore, inhibition of microglia activation is essential for recovery of neurological function after ICH. The immunohistochemistry showed that microglia activation decreased significantly after EA intervention. We considered that EA could inhibit microglia activation. Myelin sheaths are related to electrical signal transduction and nerve function [26,27]. Axonal demyelination is an important ultrastructural pathological change after ICH [28]. Under electron microscope, the degree of demyelination after EA intervention was less severe than that after ICH, indicating that EA can alleviate the damage of ICH to myelin.

As a multipotent regulator of antioxidant regulation, anti-inflammatory and microglia polarization, PPARγ has become a potential therapeutic target for stroke [29]. PPARγ can neutralize toxic substrates and reduce secondary damage by reducing oxidative stress and inflammation after ICH [30]. PPARγ can bind to PPARγ reaction element on Nrf2 promoter to promote its expression [31]. Nrf2 is a critical transcription factor for oxidative stress that binds to antioxidant response elements to promote cell survival and maintain redox homeostasis by regulating the expression of antioxidant enzymes such as catalase, GSH and SOD [32]. Under physiological conditions, Nrf2 is captured by Kelch like ECH associated protein 1 (Keap1) in cells and subsequently ubiquitinated and degraded. However, when ROS increases, the affinity of Keap1 to Nrf2 decreases, and Nrf2 is phosphorylated and translocated into the nucleus, triggering the transcription of antioxidant enzyme genes [33]. γ-GCS, as an antioxidant protein downstream of this pathway, promotes increased GSH synthesis, enhances the scavenging capacity of ROS, and can also maintain the integrity of epithelial cells and protect them from oxidative damage, exerting antioxidant capacity [34].

To sum up, our results indicate that EA may play an antioxidative role by activating PPARγ/Nrf2/γ-GCS signaling pathway, inhibiting microglia activation, and alleviating myelin injury, and then promote the repair of nerve function in rats. PPARγ blocker can attenuate the effect of EA, which further proves that EA may activate PPARγ/Nrf2/γ-GCS signaling pathway. The mechanisms of EA in treating brain injury include neuroinflammation, apoptosis and neurotrophic factors. Next, we will further explore other mechanisms by which EA improves neurological function in ICH rats.

## Acknowledgements

This work was supported by Scientific research plan project of Hebei Provincial Administration of Traditional Chinese Medicine (2024046) and Hebei Province Medical Research Project Plan (20190649).

### Conflicts of interest

There are no conflicts of interest.

## References

[1] Virani SS Alonso A Benjamin EJ Bittencourt MS Callaway CW Carson AP.; American Heart Association Council on Epidemiology and Prevention Statistics Committee and Stroke Statistics Subcommittee. Heart disease and stroke statistics-2020 update: a report from the American Heart Association. Circulation 2020; 141:e139–e596. 31992061 10.1161/CIR.0000000000000757

[2] Lan X Han X Li Q Yang QW Wang J. Modulators of microglial activation and polarization after intracerebral haemorrhage. Nat Rev Neurol 2017; 13:420–433. 28524175 10.1038/nrneurol.2017.69 PMC5575938

[3] Hanley DF Thompson RE Rosenblum M Yenokyan G Lane K Mcbee N.; MISTIE III Investigators. Efficacy and safety of minimally invasive surgery with thrombolysis in intracerebral haemorrhage evacuation (MISTIE III): a randomised, controlled, open-label, blinded endpoint phase 3 trial. Lancet 2019; 393:1021–1032. 30739747 10.1016/S0140-6736(19)30195-3 PMC6894906

[4] Fan X Xu M Ren Q Fan Y Liu B Chen J. Downregulation of fatty acid binding protein 4 alleviates lipid peroxidation and oxidative stress in diabetic retinopathy by regulating peroxisome proliferator-activated receptor gamma-mediated ferroptosis. Bioengineered 2022; 13:10540–10551. 35441580 10.1080/21655979.2022.2062533 PMC9161966

[5] Zhang L Zhang H Gu J Xu W Yuan N Sun J. Glabridin inhibits liver fibrosis and hepatic stellate cells activation through suppression of inflammation and oxidative stress by activating PPARγ in carbon tetrachloride-treated mice. Int Immunopharmacol 2022; 113:109433. 36371863 10.1016/j.intimp.2022.109433

[6] Chen H Tan H Wan J Zeng Y Wang J Wang H. Ppar-gamma signaling in nonalcoholic fatty liver disease: pathogenesis and therapeutic targets. Pharmacol Ther 2023; 245:108391. 36963510 10.1016/j.pharmthera.2023.108391

[7] Qaisiya M Coda ZC Bellarosa C Tiribelli C. Bilirubin mediated oxidative stress involves antioxidant response activation via Nrf2 pathway. Cell Signal 2014; 26:512–520. 24308969 10.1016/j.cellsig.2013.11.029

[8] Zhu Y Deng L Tang H Gao X Wang Y Guo K. Electroacupuncture improves neurobehavioral function and brain injury in rat model of intracerebral hemorrhage. Brain Res Bull 2017; 131:123–132. 28395933 10.1016/j.brainresbull.2017.04.003

[9] Chen Q Song W Tang Y Tang Y Kang Y Zhu L. Electroacupuncture reduces cerebral hemorrhage injury in rats by improving cerebral iron metabolism. Mediators Inflamm 2022; 2022:6943438. 36016663 10.1155/2022/6943438 PMC9398869

[10] Lei C Li Y Zhu X Li H Chang X. Hmgb1/tlr4 induces autophagy and promotes neuroinflammation after intracerebral hemorrhage. Brain Res 2022; 1792:148003. 35820449 10.1016/j.brainres.2022.148003

[11] Longa EZ Weinstein PR Carlson S Cummins R. Reversible middle cerebral artery occlusion without craniectomy in rats. Stroke 1989; 20:84–91. 2643202 10.1161/01.str.20.1.84

[12] Fu C Xin H Qian Z Li X Gao J Fan Y. Sinomenine protects against early brain injury by inhibiting microglial inflammatory response via Nrf2-dependent pathway after subarachnoid hemorrhage. Brain Sci 2023; 13:716. 37239188 10.3390/brainsci13050716 PMC10216249

[13] Chomiak T Hu B. What is the optimal value of the g-ratio for myelinated fibers in the rat CNS? A theoretical approach. PLoS One 2009; 4:e7754. 19915661 10.1371/journal.pone.0007754 PMC2771903

[14] Li L Wang X Guo J Chen Y Wang Z. Effect of acupuncture in the acute phase of intracerebral hemorrhage on the prognosis and serum BDNF: a randomized controlled trial. Front Neurosci 2023; 17:1167620. 37123377 10.3389/fnins.2023.1167620 PMC10133506

[15] Liu X Bao C Dong G. Using acupoint-to-acupoint penetrative needling to treat post-stroke spastic paralysis: a clinical progress review. J Tradit Chin Med 2014; 34:609–615. 25417414 10.1016/s0254-6272(15)30071-6

[16] Kuang X Fan W Hu J Wu L Yi W Lu L. Acupuncture for post-stroke cognitive impairment: a systematic review and meta-analysis. Acupunct Med 2021; 39:577–588. 34074151 10.1177/09645284211009542

[17] Zhang XH Cui H Zheng SM Lu Y Yuan HW Zhang L. Electroacupuncture regulates microglial polarization via inhibiting NF-κB/COX2 pathway following traumatic brain injury. Brain Res 2023; 1818:148516. 37562566 10.1016/j.brainres.2023.148516

[18] Guan R Li Z Dai X Zou W Yu X Liu H. Electroacupuncture at gv20-gb7 regulates mitophagy to protect against neurological deficits following intracerebral hemorrhage via inhibition of apoptosis. Mol Med Rep 2021; 24:492. 33955500 10.3892/mmr.2021.12131 PMC8127033

[19] Deng L Zhou L Zhu Y Fan G Tang H Zheng Y. Electroacupuncture enhance therapeutic efficacy of mesenchymal stem cells transplantation in rats with intracerebral hemorrhage. Stem Cell Rev Rep 2022; 18:570–584. 33661471 10.1007/s12015-021-10144-8

[20] Cheng Y Zan J Song Y Yang G Shang H Zhao W. Evaluation of intestinal injury, inflammatory response and oxidative stress following intracerebral hemorrhage in mice. Int J Mol Med 2018; 42:2120–2128. 30015849 10.3892/ijmm.2018.3755

[21] Zhang Y Khan S Liu Y Wu G Yong VW Xue M. Oxidative stress following intracerebral hemorrhage: from molecular mechanisms to therapeutic targets. Front Immunol 2022; 13:847246. 35355999 10.3389/fimmu.2022.847246 PMC8959663

[22] Chen S Li L Peng C Bian C Ocak PE Zhang JH. Targeting oxidative stress and inflammatory response for blood-brain barrier protection in intracerebral hemorrhage. Antioxid Redox Signal 2022; 37:115–134. 35383484 10.1089/ars.2021.0072

[23] Michell-Robinson MA Touil H Healy LM Owen DR Durafourt BA Bar-Or A. Roles of microglia in brain development, tissue maintenance and repair. Brain 2015; 138:1138–1159. 25823474 10.1093/brain/awv066 PMC5963417

[24] Aguilar MI Freeman WD. Spontaneous intracerebral hemorrhage. Semin Neurol 2010; 30:555–564. 21207348 10.1055/s-0030-1268865

[25] Liu F Lu J Manaenko A Tang J Hu Q. Mitochondria in ischemic stroke: new insight and implications. Aging Dis 2018; 9:924–937. 30271667 10.14336/AD.2017.1126 PMC6147588

[26] Zhang Q Zhu W Xu F Dai X Shi L Cai W. The interleukin-4/PPARγ signaling axis promotes oligodendrocyte differentiation and remyelination after brain injury. PLoS Biol 2019; 17:e3000330. 31226122 10.1371/journal.pbio.3000330 PMC6608986

[27] Mckenzie IA Ohayon D Li H de Faria JP Emery B Tohyama K. Motor skill learning requires active central myelination. Science 2014; 346:318–322. 25324381 10.1126/science.1254960 PMC6324726

[28] Li Q Weiland A Chen X Lan X Han X Durham F. Ultrastructural characteristics of neuronal death and white matter injury in mouse brain tissues after intracerebral hemorrhage: coexistence of ferroptosis, autophagy, and necrosis. Front Neurol 2018; 9:581. 30065697 10.3389/fneur.2018.00581 PMC6056664

[29] Zhao XR Gonzales N Aronowski J. Pleiotropic role of PPARγ in intracerebral hemorrhage: an intricate system involving Nrf2, RXR, and NF-κB. CNS Neurosci Ther 2015; 21:357–366. 25430543 10.1111/cns.12350 PMC4376579

[30] Itoh K Wakabayashi N Katoh Y Ishii T O’Connor T Yamamoto M. Keap1 regulates both cytoplasmic-nuclear shuttling and degradation of Nrf2 in response to electrophiles. Genes Cells 2003; 8:379–391. 12653965 10.1046/j.1365-2443.2003.00640.x

[31] Park EY Cho IJ Kim SG. Transactivation of the PPAR-responsive enhancer module in chemopreventive glutathione S-transferase gene by the peroxisome proliferator-activated receptor-gamma and retinoid X receptor heterodimer. Cancer Res 2004; 64:3701–3713. 15150131 10.1158/0008-5472.CAN-03-3924

[32] Yao H He Q Huang C Wei S Gong Y Li X. Panaxatriol saponin ameliorates myocardial infarction-induced cardiac fibrosis by targeting Keap1/Nrf2 to regulate oxidative stress and inhibit cardiac-fibroblast activation and proliferation. Free Radic Biol Med 2022; 190:264–275. 35977659 10.1016/j.freeradbiomed.2022.08.016

[33] Kopacz A Kloska D Forman HJ Jozkowicz A Grochot-Przeczek A. Beyond repression of Nrf2: an update on keap1. Free Radic Biol Med 2020; 157:63–74. 32234331 10.1016/j.freeradbiomed.2020.03.023 PMC7732858

[34] Zhu X Zhang YM Zhang MY Chen YJ Liu YW. Hesperetin ameliorates diabetes-associated anxiety and depression-like behaviors in rats via activating Nrf2/are pathway. Metab Brain Dis 2021; 36:1969–1983. 34273043 10.1007/s11011-021-00785-6

